# Changes in ADAR RNA editing patterns in CMV and ZIKV congenital infections

**DOI:** 10.1186/s12864-023-09778-4

**Published:** 2023-11-15

**Authors:** Benjamin Wales-McGrath, Heather Mercer, Helen Piontkivska

**Affiliations:** 1 University of Pennsylvania, Perelman School of Medicine, Department of Genetics, Philadelphia, PA USA; 2 Children’s Hospital of Philadelphia, Division of Cancer Pathobiology, Philadelphia, PA USA; 3 Department of Biological and Environmental Sciences, University of Mount Union, Alliance, OH USA; 4 Department of Biological Sciences, Kent State University, Kent, OH USA; 5 School of Biomedical Sciences, Kent State University, Kent, OH USA; 6 Brain Health Research Institute, Kent State University, Kent, OH USA; 7 Healthy Communities Research Institute, Kent State University, Kent, OH USA

**Keywords:** RNA editing, Adenosine deaminases acting on RNA (ADAR) editing, A-to-I editing, Immune response, Neurodevelopment, Zika virus, Cytomegalovirus, Congenital infection, Maternal immune activation (MIA)

## Abstract

**Background:**

RNA editing is a process that increases transcriptome diversity, often through Adenosine Deaminases Acting on RNA (ADARs) that catalyze the deamination of adenosine to inosine. ADAR editing plays an important role in regulating brain function and immune activation, and is dynamically regulated during brain development. Additionally, the ADAR1 p150 isoform is induced by interferons in viral infection and plays a role in antiviral immune response. However, the question of how virus-induced ADAR expression affects host transcriptome editing remains largely unanswered. This question is particularly relevant in the context of congenital infections, given the dynamic regulation of ADAR editing during brain development, the importance of this editing for brain function, and subsequent neurological symptoms of such infections, including microcephaly, sensory issues, and other neurodevelopmental abnormalities. Here, we begin to address this question, examining ADAR expression in publicly available datasets of congenital infections of human cytomegalovirus (HCMV) microarray expression data, as well as mouse cytomegalovirus (MCMV) and mouse/ human induced pluripotent neuroprogenitor stem cell (hiNPC) Zika virus (ZIKV) RNA-seq data.

**Results:**

We found that in all three datasets, ADAR1 was overexpressed in infected samples compared to uninfected samples. In the RNA-seq datasets, editing rates were also analyzed. In all mouse infections cases, the number of editing sites was significantly increased in infected samples, albeit this was not the case for hiNPC ZIKV samples. Mouse ZIKV samples showed altered editing of well-established protein-recoding sites such as Gria3, Grik5, and Nova1, as well as editing sites that may impact miRNA binding.

**Conclusions:**

Our findings provide evidence for changes in ADAR expression and subsequent dysregulation of ADAR editing of host transcriptomes in congenital infections. These changes in editing patterns of key neural genes have potential significance in the development of neurological symptoms, thus contributing to neurodevelopmental abnormalities. Further experiments should be performed to explore the full range of editing changes that occur in different congenital infections, and to confirm the specific functional consequences of these editing changes.

**Supplementary Information:**

The online version contains supplementary material available at 10.1186/s12864-023-09778-4.

## Background

Congenital infections of an unborn fetus or newborn infant are a widespread issue caused by pathogens such as cytomegalovirus (CMV) and Zika virus (ZIKV) [1, 2]. These viruses frequently disrupt fetal brain development, resulting in a wide range of symptoms, including damage to sensory organs, microcephaly and other central nervous system (CNS) abnormalities, or learning and behavioral disorders [1, 3–9]. This is a result of the neural tropism of both CMV [10] and ZIKV [11–13], resulting in the infection and dysregulation of neural stem cells (NSCs) and neural progenitor cells (NPCs) [13–15]. Interestingly, neurological dysregulation can occur as an indirect effect of the antiviral immune response, as has been observed in CMV [16–18], ZIKV [19, 20], and other congenital infections [1, 14, 21]. However, the mechanisms by which infections cause these effects remain to be fully elucidated and can be highly dependent on the stage of pregnancy where infection occurs and other context-dependent factors [1, 14, 22].

ADAR enzymes are the primary cause of RNA editing, catalyzing the deamination of adenosine to inosine in dsRNA. The ADAR family contains three gene loci in mammals: ADAR1, ADAR2, and ADAR3 [23–25]. Of these, only ADAR1 and 2 are known to engage in editing, as ADAR3 is catalytically inactive, and it is believed to inhibit editing by ADAR1 and 2 [26–28]. ADAR1 has two isoforms (p110 and p150), which are transcribed from unique promoters, the latter of which is interferon (IFN) inducible due to an IFN-sensitive response element (ISRE) [29, 30]. In addition, ADAR1p110 and ADAR2 are primarily localized to the nucleus, while ADAR1 p150 can be exported to the cytoplasm, a factor which may contribute to differences between their targets [24, 26]. Editing by these enzymes can take the form of highly selective site-specific editing or less selective hyper-editing of multiple bases in a single transcript, usually preferring double stranded regions [24, 31]. Most of these editing sites occur in non-coding regions of transcripts, primarily in transcripts of repetitive sequences such as Alu sequences in humans or SINE sequences in mice, as well as in introns and 3’UTRs [32]. However, a small, but disproportionately important minority of editing sites occur in protein coding RNA sequences [26, 30].

ADAR editing can affect protein coding, as well as splicing, miRNA binding, and other non-coding RNAs. While the number of protein recoding editing sites is relatively small, many of these sites are highly conserved and have physiologically significant effects, including in neurologically significant genes such as GRIA2, HTR2C, and NOVA1 [26, 33–36]. Dysregulation of this editing has been linked to neurological diseases, including neurodegeneration and psychiatric disorders [37–41]. Additionally, ADAR editing can disrupt mRNA splicing by recoding splice sites or splicing regulatory elements (SREs) [24, 42–44]. ADAR also regulates RNA interference through diverse mechanisms, including by editing of miRNAs or miRNA binding sites in 3′ UTRs, as well as editing-independent interactions with Dicer which effect miRNA processing [26, 45–48]. Overall, regulation of ADAR editing in the brain is complex, with nuanced changes over development, across brain regions, and in different disease states, and with a dynamic interplay between different ADAR enzymes which remains to be fully understood [37, 49, 50].

Aside from its function in gene regulation, ADAR enzymes (primarily ADAR1 p150 isoform) play a dual role in the antiviral innate immune response. As noted previously, expression of ADAR1 p150 is induced by signaling from type 1 IFNs, a class of cytokine secreted in response to viral infection [51, 52]. During the antiviral immune response, editing has complex pro- or anti-viral effects, dependent on host/virus-specific factors or even changing over the course of infection. The variable effects of this editing can disrupt the function of viral proteins, while suppressing interferon responses or potentially giving rise to variants that enable immune escape [23, 53]. Evidence of ADAR1 p150 activation by IFNs leads to the question: what impact does the induction of ADAR1 p150 expression in response to viral infection have on normal host transcriptome editing? This question is particularly significant in the brain, and in the context of congenital infection, given the importance of proper regulation of ADAR editing for brain development. However, a handful of prior studies showed conflicting results as to the effect of infection on ADAR editing rates [reviewed in 23]. While infection of primary human neural stem cells with Zika virus has been shown to cause increased ADAR editing (including in a GRIA3 recoding site) [54], infection of neonatal mice by a neurotropic strain of reovirus (ReoV) showed that, despite a strong induction of ADAR1 p150, editing changes were limited to a small number of sites [55]. Interestingly, maternal immune activation (MIA) using poly(I:C) to induce the interferon response in pregnant mice has also been shown to result in increases in changes in ADAR expression and editing, resulting in long term neurodevelopmental symptoms later in life, despite transient editing changes [56].

Here, we explore whether congenital viral infection is associated with changes in ADAR editing of key host genes, and whether genes with editing changes can be linked to important neurodevelopmental functions, by examining publicly available microarray data of congenital human cytomegalovirus (HCMV) infection and RNA-seq data of congenital mouse cytomegalovirus (MCMV) infection and Zika virus (ZIKV) infection in mice to assess the effect of congenital infections on ADAR expression and editing. Our results show increased ADAR1/2 expression and increased A-to-I editing associated with infection, including changes in editing in genes relevant to neurodevelopment, providing support for the hypothesis that dysregulation of ADAR editing contributes to neurodevelopmental abnormalities caused by congenital infection.

## Results

To examine the effects of congenital infections on ADAR editing in the developing brain, we examined transcriptome datasets from humans or model organisms with congenital infections. Using the NCBI BioProject database, we identified 5 such relevant datasets with humans and mice infected with ZIKV and CMV, as described in Table 1.

**Table 1:** List of BioProject datasets of viral infections used in this study, and their characteristics

BioProject Accession	Virus	Time of Infection	Organism	Virus Delivery	Tissue	ADAR1 Expression	ADAR2 Expression	ADAR3 Expression	Editing Site Number	Editing Sites with Increased Editing	Editing Sites with Decreased Editing	
PRJNA422858	HCMV^#^	Infants at time of diagnosis	Human	Natural	Blood	Up (p_adj = 7.31E-07)	Up (p_adj = 7.59E-05 and 4.00E-03)	No Change	–	–	–	–
PRJEB38849	MCMV	Newborn mice, analyzed after 8 days infection	Mouse	inoculated i.p. with 400 PFU	Microglia	Up (p_adj < 0.001)	No Change	No Change	Up (p = 0.01129)	21	0	Up (p = 0.0004)
PRJNA487357	ZIKV	E10.5/E14.5	Mouse	unspecified	Brain	Up (p_adj < 0.001)	No Change	No Change	Up (p = 0.01877)	12	0	Up (p = 0.004)
PRJNA358758	ZIKV	E15.5/P3	Mouse	6.5 × 105 PFU injected into one side of the lateral ventricle (LV)	Brain	Up (p_adj < 0.001)	Down (p_adj < 0.001)	Down (p_adj < 0.001)	Up (*p* = 0.0007137)	137	11	Up (p = 0.006)
PRJNA551246	ZIKV	Analysis after 48 hours infection	Human	Inoculated for 1 hr. with virus at MOI 1	hiNPC	Higher in PE243V (p_adj < 0.001) and FSS1302 (p_adj < 0.001) than control; higher in FSS1302 than PE243V (p_adj < 0.001)	No Change	No Change	No Change	3 in FSS1302; 1 in PE243V; 1 in both	2 in FSS1302; 1 in PE243V; 1 higher in PE243V than FSS1302	Increased in both ZIKV strains (p < 0.0001), no change between ZIKV strains

Characteristics of BioProject datasets of infections of Zika virus, ZIKV, and human and mouse cytomegalovirus, HCMV and MCMV, respectively

Abbreviations: i.p intraperitoneal injection, PFU plaque-forming unit, MOI multiplicity of infection, p_adj *p* value adjusted for multiple testing

^#^ HCMV expression values are from the symptomatic samples compared to controls. ADAR2 values are from two different probes, ILMN_1679797 and ILMN_2319326, respectively

### HCMV gene expression analysis

In order to understand the effects of congenital HCMV infection, we used data available in BioProject PRJNA422858. The original analysis [57] used blood samples of infants with symptomatic and asymptomatic congenital HCMV infections and performed microarray analysis to obtain gene expression data. While this dataset did not allow us to directly evaluate editing rates due to the lack of sequencing data and resultant inability to perform variant calling, it provided a large, clinical sample to evaluate the changes in expression of ADAR genes caused by congenital HCMV infection.

Reanalyzing the data using the NCBI GEO2R tool [58], both ADAR1 and ADAR2 were found to be significantly overexpressed in HCMV samples compared to controls. Specifically, both genes were overexpressed in symptomatic vs. control HCMV samples (p-adj of 7.31E-07, and 7.59E-05 and 4.00E-03 for ADAR1 p150 probe ILMN_1776777, ADAR2 probes ILMN_1679797 and ILMN_2319326, respectively), and asymptomatic vs. control HCMV samples (p-adj of 3.54E-04, and 6.34E-05 and 1.62E-02, for probes ILMN_1776777, ILMN_1679797 and ILMN_2319326, respectively) (Supplementary File 1). However, no differences were detected between asymptomatic and symptomatic HCMV samples (p-adj of 0.338, and 0.874 and 0.981 for probes ILMN_1776777, ILMN_1679797 and ILMN_2319326, respectively). In addition to this, Reactome pathway analysis [59] was used to explore pathways experiencing differential gene expression. Both asymptomatic and symptomatic HCMV samples showed an enrichment in IFN signaling pathways among differentially expressed genes, including Interferon alpha/beta signaling linked to ADAR1 p150 expression (p-adj = 4.48E-04 and 0.001747396, and 0.005521773 and 0.010567466 for Interferon alpha/beta signaling (R-HSA-909733) and Interferon Signaling (R-HSA-913531) pathways in asymptomatic and symptomatic samples, respectively). Full lists of differentially expressed genes and significantly overrepresented Reactome pathways are listed in the Supplementary File 1.

### MCMV RNA-seq data analysis

**Fig. 1:**
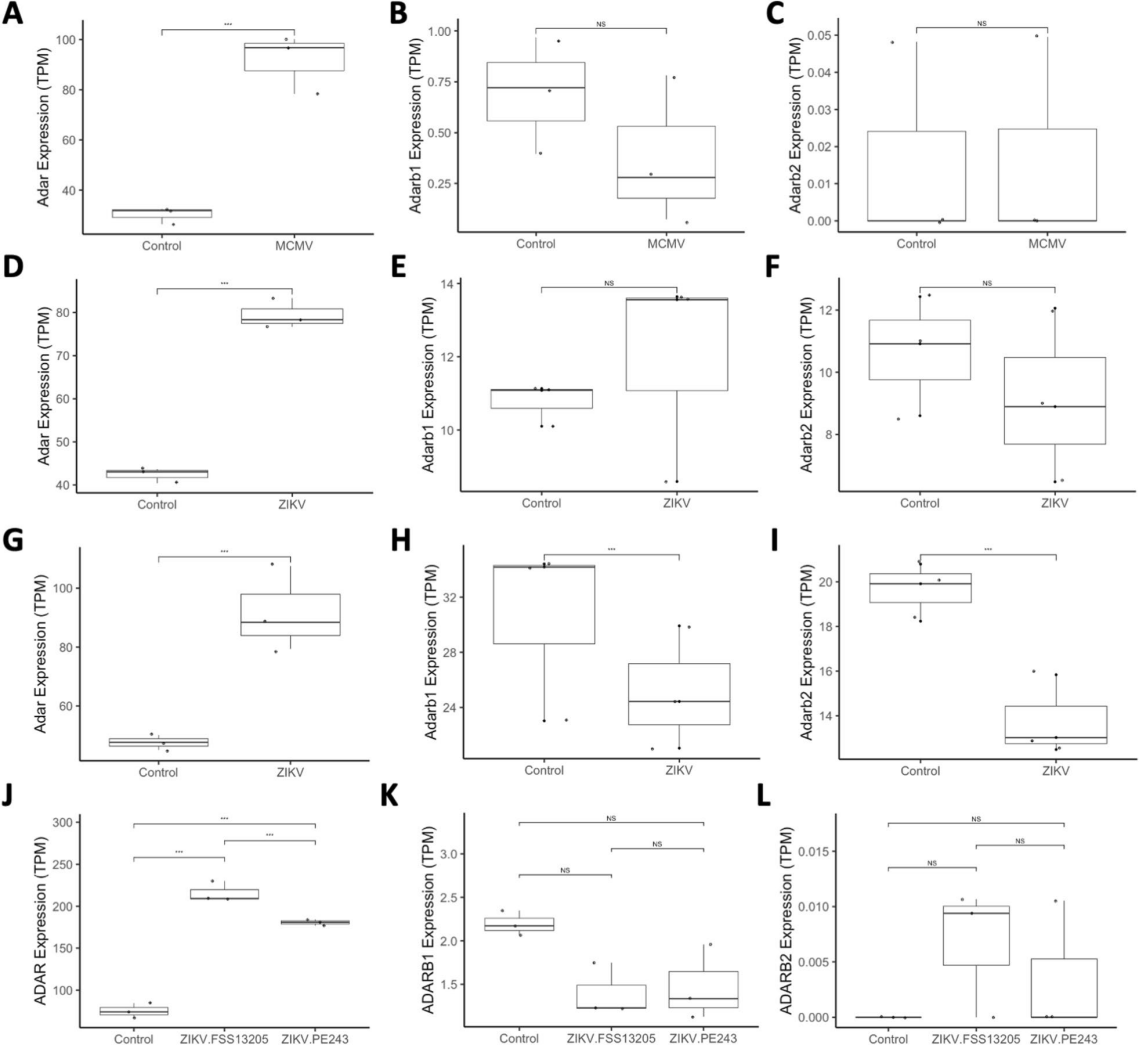
Box plots of ADAR1 (ADAR), ADAR2 (ADARb1) and ADAR3 (ADARb2) expression, in transcripts per million (TPM). Panels A, B and C depict expression differences between control vs. MCMV-infected mice samples. Only ADAR1 expression differed significantly between control and infected samples (p_adj_ < 0.001), but not that of ADAR2 or ADAR3 (p_adj_ > 0.05). Panels D, E and F show expression in control vs. ZIKV-infected samples (PRJNA487357). Similar to MCMV infections, only ADAR1 expression differed significantly between control and infected samples (p_adj_ < 0.001), but not that of ADAR2 or ADAR3 (p_adj_ > 0.05). Panels G, H and I show expression in control vs. ZIKV-infected samples (PRJNA358758). Expression of all three genes differed significantly between control and infected samples (p_adj_ < 0.001), albeit in different directions (ADAR1 was over-, while ADAR2 and ADAR3 were under-expressed, respectively). Panels J, K and L show expression in control vs. ZIKV-infected human induced pluripotent neuroprogenitor stem cells (hiNPCs) samples (PRJNA551246). Infections with Cambodian (FSS13025) and Brazilian ZIKV (PE243) strains are shown separately. ADAR1 expression differed significantly between control and infected samples for both strains, and was also higher in FSS13025-infected samples compared to PE243V-infected ones (p_adj_ < 0.001 in all these comparisons). ADAR2 and ADAR3 expression did not differ significantly between conditions (p_adj_ > 0.05)

Next, we examined available RNA-seq data to investigate the effects of mouse cytomegalovirus (MCMV). The original dataset [60] performed RNA-seq of the microglia of 3 newborn mice infected with MCMV and 3 controls after 8 days of infection. Using DESeq2 differential gene expression analysis [61], we found that ADAR1 was overexpressed in infected samples (log_2_FC = 1.79839, p_adj_ < 0.001), as shown in Fig. 1 A, while ADAR2/3 were not significantly changed (Fig. 1 B-C, respectively). Transcript-level analysis showed that ADAR1 overexpression was due to overexpression of ADAR p150 but not p110 isoform (p_adj_ = 0.0088 vs 0.65, respectively, with log_2_FC = 1.51 for p150) (Supplementary File 2A-3A).

**Fig. 2:**
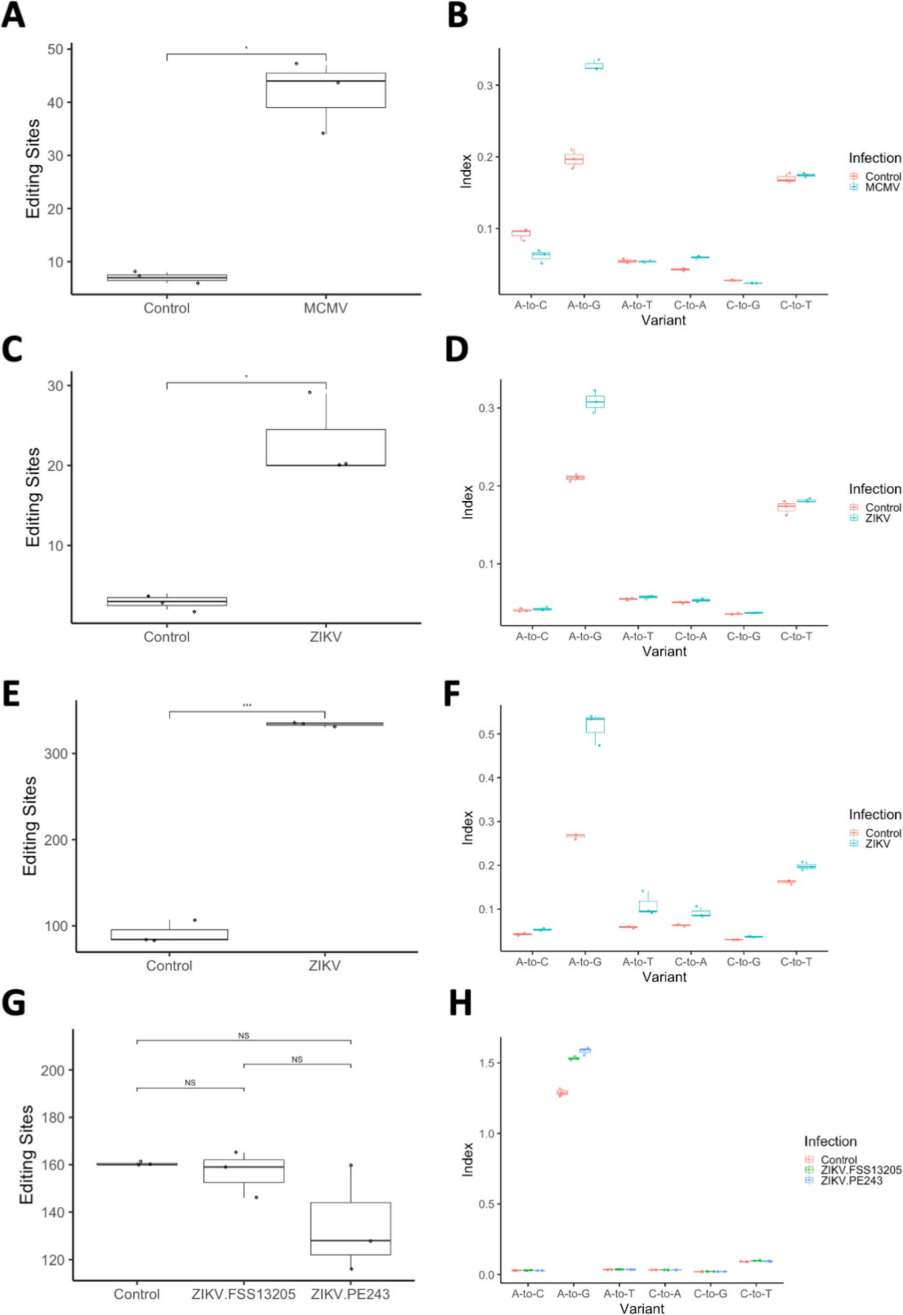
ADAR editing differences between control and infected samples, shown as the box plots of the number of editing sites and the Alu/SINE Editing Index (AEI). Panels A and B show the editing sites and AEI, respectively, between control vs. MCMV-infected mice samples. Both differed significantly between control vs. MCMV-infected mice (*p* = 0.01129 and *p* = 0.0004, respectively). Panels C and D show the editing sites and AEI, respectively, between control vs. ZIKV-infected samples (PRJNA487357). Both the number of editing sites and the average editing rates differed significantly between control vs. ZIKV-infected samples (*p* = 0.01877 and *p* = 0.004, respectively). Panels E and F show the editing sites and AEI, respectively, between control vs. ZIKV-infected samples (PRJNA358758). Both the number of editing sites and AEI differed significantly between control vs. ZIKV-infected samples (*p* = 0.0007123 and *p* = 0.006, respectively). Panels G and H show the editing sites and AEI, respectively, between ZIKV-infected human induced pluripotent neuroprogenitor stem cells (hiNPCs) samples (PRJNA551246). Infections with Cambodian (FSS13025) and Brazilian ZIKV (PE243) strains are shown separately. Unlike other ZIKV-infection examples, no significant differences were detected in the number of editing sites, but both ZIKV strains had significantly higher AEI than controls (*p* < 0.0001), with no significant difference between strains

**Fig. 3:**
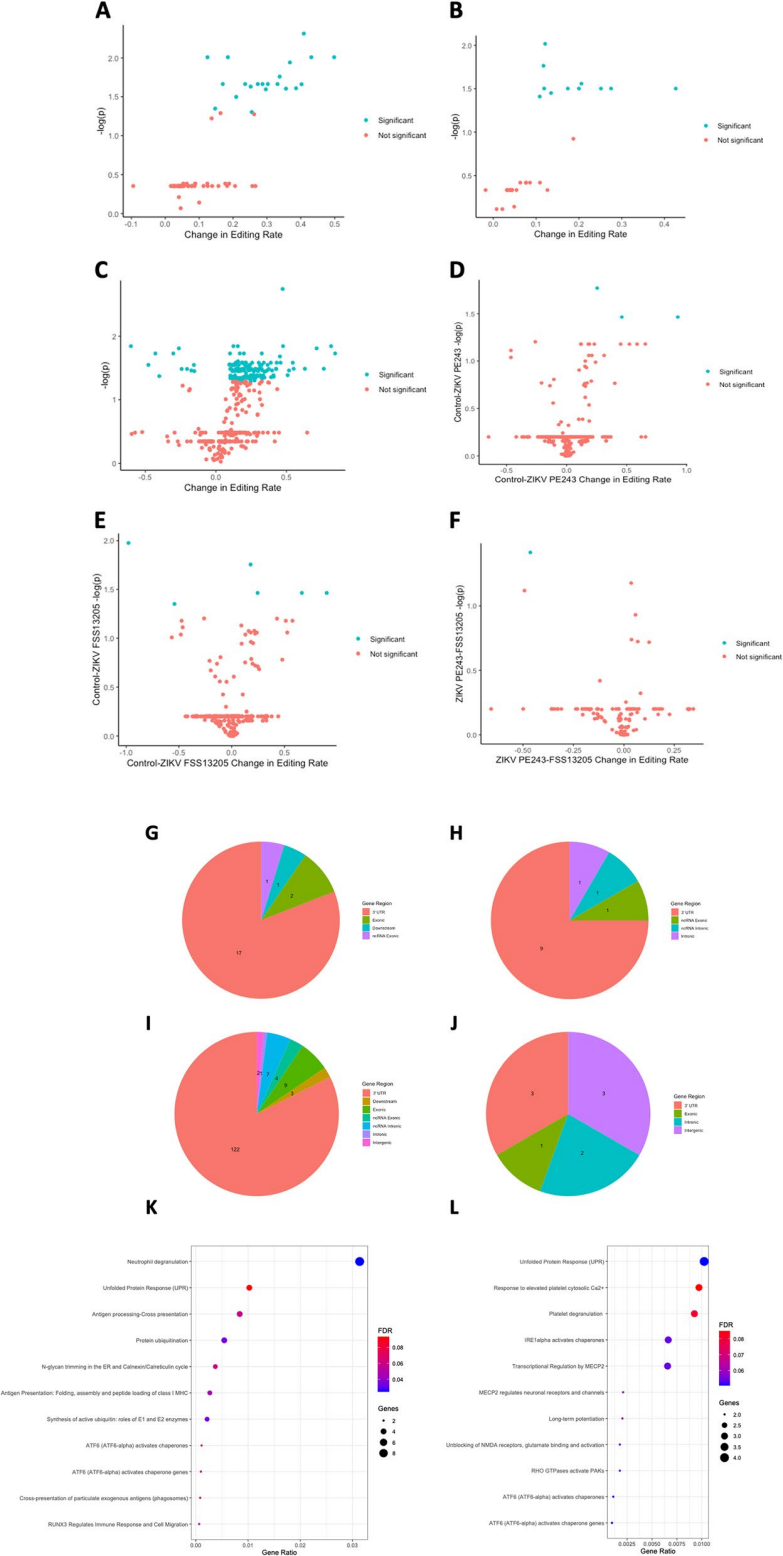
Features of significantly changing site-specific editing, including volcano plots, gene regions of editing sites, and pathway enrichment analysis results. Panel A: Volcano plot of magnitude of changes in editing rate vs log transformed *p* value (FDR adjusted) of changes in editing rate for MCMV vs control samples (see Supplementary File 5B). 21 sites see significant increases in editing, with none significantly decreasing, p_adj < 0.05 (shown in blue). Panel B: Volcano plot of magnitude of changes in editing rate vs log transformed p value (FDR adjusted) of changes in editing rate for ZIKV vs control samples in PRJNA487357 (see Supplementary File 7B). 12 sites see significant increases in editing, with none significantly decreasing, p_adj < 0.05 (shown in blue). Panel C: Volcano plot of magnitude of changes in editing rate vs log transformed p value (FDR adjusted) of changes in editing rate for ZIKV vs control samples in PRJNA358758 (see Supplementary File 8B). 137 sites see significant increases in editing, while 11 see significant decreases, p_adj < 0.05 (shown in blue). Panel D: Volcano plot of magnitude of changes in editing rate vs log transformed p value (FDR adjusted) of changes in editing rate for ZIKV PE243 vs control hiNPC samples (see Supplementary File 10B). 3 sites see significant increases in editing, while none see significant decreases, p_adj < 0.05 (shown in blue). Panel E: Volcano plot of magnitude of changes in editing rate vs log transformed p value (FDR adjusted) of changes in editing rate for ZIKV FSS13205 vs control samples (see Supplementary File 10B). 4 sites see significant increases in editing, while 1 sees a significant decrease, p_adj < 0.05 (shown in blue). Panel F: Volcano plot of magnitude of changes in editing rate vs log transformed p value (FDR adjusted) of changes in editing rate for ZIKV FSS13205 vs PE243 samples (see Supplementary File 10B). One site sees a significant decrease in editing, p_adj < 0.05 (shown in blue). Panels G-J: Pie chart of the gene regions of significantly changing editing sites in MCMV, ZIKV PRJNA487357, ZIKV PRJNA358758, and ZIKV hiNPC data, respectively. Panels K-L: Dot plots showing significantly enriched Reactome pathways for editing sites (FDR < 0.1) in MCMV and ZIKV PRJNA487357 samples, respectively. Color indicates FDR, size of dot indicates the number of sites per pathway, and X axis shows gene ratio of the pathway enriched. ZIKV PRJNA358758 did not have any significantly enriched pathways (FDR < 0.1) and was not plotted

To determine the effect of this on global editing levels, we used the *Alu* editing index (AEI) method [62], which calculates the ratio of A-to-G mismatches to total A coverage in repetitive regions affected by hyperediting (Alu elements in humans, or B1/B2 SINE elements in mice). Using this approach, we found a significant increase in A-to-G hyperediting in MCMV-infected samples compared to controls, *p* = 0.0004 (Fig. 2 B; Supplementary File 4A). Specifically, high-confidence editing events were detected 149 times across MCMV and control samples at 69 genomic sites (Supplementary File 5A). Moreover, editing sites were disproportionately found in MCMV-infected samples, as shown in Fig. 2 A (*p* = 0.01129). Interestingly, Supplementary Fig. 1 shows that the range of editing rates is much wider in MCMV samples than controls. Site-specific analysis showed 21 sites with significantly increased editing in MCMV samples, with no sites showing significant decreases in editing (Fig. 3 A; Supplementary File 5B), with the majority of changes occurring in 3′ UTR regions (Fig. 3 G). This included three editing sites in the Lamp2 gene, a membrane glycoprotein involved in lysosomal functions, a site in the Kcnk6 potassium channel, and a site in Tmem9b, which enhances proinflammatory cytokine production (full list is available in Supplementary File 5B). Reactome pathway analysis of sites edited in MCMV infection showed overrepresentation (FDR < 0.1) among immune-related pathways (Supplementary File 6A; Fig. 3 K). Notably, some of the editing targets, such as Cybb and Arhgdia, belong to multiple pathways, including neurologically relevant ones, such as RAC1 GTPase cycle (R-HSA-9013149), involved in neuronal development [63].

### Mouse ZIKV RNA-seq data analysis

We next wanted to investigate the effect of ZIKV on ADAR editing. To do this, we first used two RNA-seq datasets of ZIKV-infected mice. The first of these datasets (BioProject PRJNA487357), was derived from whole brains of 3 ZIKV infected and 3 control mouse embryos at E14.5 after infection at E10.5. Again, we found ADAR1 was overexpressed in infected samples (log_2_FC = 0.801943, p_adj_ < 0.001) (Fig. 1 D), while ADAR2 and 3 were not significantly affected (Fig. 1 E-F), though transcript-level analysis for ADAR1 was not possible due to low coverage (Supplementary File 2B-3B). The AEI was once again shown to be significantly increased in ZIKV-infected samples (*p* = 0.004), showing increased levels of global, non-specific editing (Fig. 2 D; Supplementary File 4B). High confidence editing events were detected 78 times across ZIKV and control samples at 34 genomic coordinates (Supplementary File 7A), disproportionately found in ZIKV samples, as shown in Fig. 2 C (*p* = 0.01877).

A site-specific analysis showed that most sites experienced increased editing in ZIKV samples, and none with decreased editing (Supplementary File 7B; Fig. 3 B). Similar to MCMV samples, the majority of editing changes were found in 3′ UTR regions (Fig. 3 H). Interestingly, Gria2 was edited in both conditions with no significant difference in editing rate, while Blcap and Snhg11 showed higher levels of editing in ZIKV samples. Reactome pathway analysis of editing targets showed overrepresentation (FDR < 0.1) of several neurologically relevant pathways, including “unblocking of NMDA receptors, glutamate binding and activation,” “long-term potentiation,” “MECP2 regulates neuronal receptors and channels,” and “transcriptional regulation by MECP2”. The latter two pathways are noteworthy due to prominent role of MECP2 in modulating synaptic plasticity [64, 65] (Supplementary File 6B; Fig. 3 L).

The second mouse ZIKV dataset [19] consisted of RNA-seq of the whole brains of 3 ZIKV SZ01 infected and 3 control embryonic mouse P3 brains infected at E15.5. ADAR was found to be overexpressed in ZIKV samples (log_2_FC = 0.654311, p_adj_ < 0.001), while ADAR2 (log_2_FC = − 0.570186, p_adj_ < 0.001) and ADAR3 (log_2_FC = − 0.779223, p_adj_ < 0.001) were underexpressed in ZIKV samples (Fig. 1 G, H, I, respectively). Transcript-level analysis for ADAR1 showed that both p150 and p110 isoforms were upregulated (p_adj_ = 0.0049 and 0.00047, respectively, and log_2_FC = 0.63 and 0.90 respectively) (Supplementary File 2C-3C). As a result, the AEI was found to be significantly increased in infected samples, *p* = 0.006 (Fig. 2 F; Supplementary File 4C). Notably, this dataset has a significantly higher read depth than the previously analyzed RNA-seq data, with 48–65 million reads, compared to 16–25 million and 10–16 million reads in PRJEB38849 and PRJNA487357 respectively. This allowed detection of a much greater number of high confidence editing sites, as editing was detected a total of 1276 times in total at 501 genomic coordinates (Supplementary File 8A), likewise disproportionately found in ZIKV samples (*p* = 0.0007123) (Fig. 2 E, F).

Numerous edited sites harbored significant differences in editing, including 11 sites with significantly decreased editing in ZIKV samples and 137 sites with significantly increased editing in ZIKV samples (Supplementary File 8B; Fig. 3 C), once again mostly in 3′ UTRs (Fig. 3 I). This included 9 sites in exonic regions, including key neural genes such as Gria3, Grik5, and Nova1. While members of many of the same neural-related pathways were identified among edited targets, there were no overrepresented pathways that passed the FDR < 0.1 cut-off threshold in Reactome pathway analysis (Supplementary File 6C). Significantly, there was a high degree of overlap between the two mouse ZIKV datasets, as 9 of 12 coordinates and 9 of 10 genes with differential editing in PRJNA487357 were also differentially edited in PRJNA358758.

**Fig. 4:**
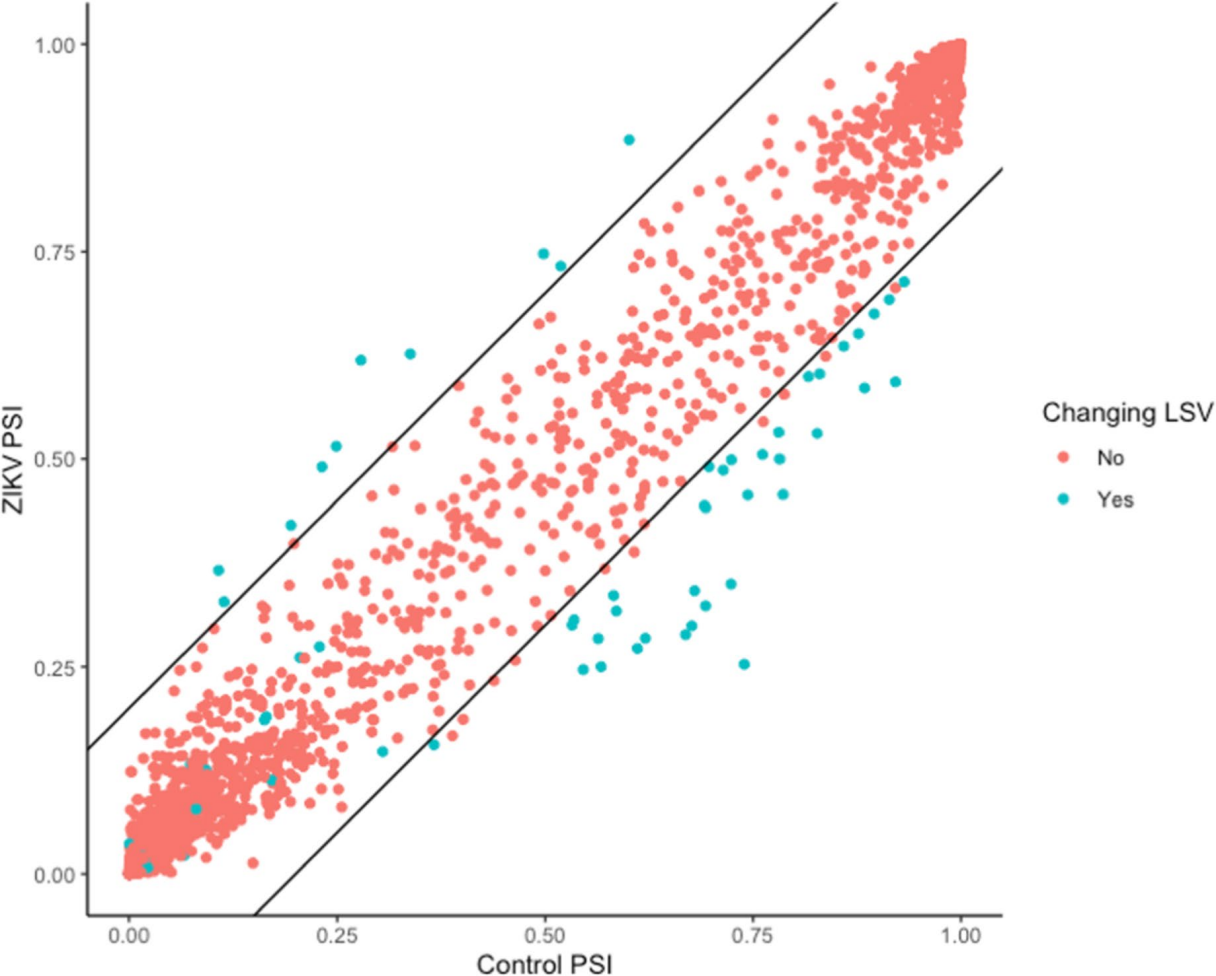
Scatterplot showing splicing changes between ZIKV and Control samples for PRJNA358758 for all LSVs in genes targeted by Nova1, as per Zhang et al., 2010. Significantly changing local splicing variations, LSVs P(|dPSI| > 0.2) > 0.95 are shown in blue, with lines (slope = 1 and y intercept = − 0.2/0.2) to show 0.2 change in percent spliced in (PSI) values

Due to the finding of differential editing in Nova1 and other splicing factors, we evaluated local splicing variations (LSVs) between infected and uninfected samples using MAJIQ and VOILA [66], identifying 936 LSVs with high confidance changes in percent spliced in (PSI) greater than 20% in 650 genes (Fig. 4; Supplementary File 9). Of these, 87 LSVs in 49 genes occurred in genes identified as Nova targets as per [67], indicating dysregulation of Nova RNA splicing. Specifically, 25 LSVs were detected in 13 disease-associated Nova targets, indicating the potential significance of this dysregulation. However, it is unclear whether this dysregulation is related to changes in Nova1 editing by ADAR.

### hiNPC ZIKV RNA-seq data analysis

We further analyzed a dataset of human induced pluripotent neuroprogenitor stem cells (hiNPCs), allowing us to look specifically at editing in NPCs and to get a perspective from human samples. The original study [20] took RNA-seq data from hiNPCs; 3 controls, 3 infected with the Cambodian strain of ZIKV (FSS13025), and 3 infected with the Brazilian strain of ZIKV (PE243). The AEI was significantly increased in both the FSS13205 and PE243 strains of ZIKV compared to controls (*p* < 0.0001), but not significantly different between strains (Fig. 2 H; Supplementary File 4D). In these samples, high confidence editing was detected 1355 times in total at 312 genomic coordinates (Supplementary File 10A). ADAR1 expression was found to be higher in samples infected with both PE243V and FSS13025 ZIKV (log_2_FC = 1.45412, p_adj_ < 0.001 and log_2_FC = 1.79357, p_adj_ < 0.001), and was also found to be higher in samples infected with the FSS13025 strain compared to the PE243V strain (log_2_FC = 0.339453, p_adj_ < 0.001) (Fig. 1 J). Specifically, ADAR1 p150 was upregulated in both ZIKV strains compared to controls, and also higher in the FSS13025 strain than the PE243 strain. ADAR1 p110 was also upregulated in both strains compared to controls, but not significantly different between strains (Supplementary File 2E-F and 3E-F). However, ADAR2 and ADAR3 had no significant changes in expression (Fig. 1 K, L). Unlike previous samples, no significant differences were detected in the number of editing sites, but some ZIKV samples had a lower number of editing sites than the control samples (Fig. 2 G, H; Supplemental File 10A).

Nonetheless, analysis of differences at specific editing sites found 9 sites with significantly different editing rates between conditions (Supplementary File 10B; Fig. 3 D, E, F, J). Of these sites, 3 were increased and 2 were decreased in ZIKV FSS1302 compared to controls, one was increased in ZIKV PE243V compared to controls, one was increased in both ZIKV FSS1302 and PE243V compared to controls, and one was increased in ZIKV PE243V compared to ZIKV FSS1302. Specifically, all three editing sites which saw increased editing in ZIKV FSS1302 vs controls were in the DDX58 gene, which codes for the RIG-1 receptor. Additionally, B9D1, a gene involved with ciliogenesis, is almost completely edited in control samples, but only edited in one ZIKV PE243V sample and no ZIKV FSS1302 samples. This gene is implicated in the development of Meckel Syndrome, characterized by CNS developmental abnormalities, such as encephalocele [68, 69]. In both ZIKV FSS1302 and PE243V samples, an exonic site in the IFITM2 gene which is not edited in controls is highly edited (between 88 and 96%). IFITM2 is an IFN-induced transmembrane gene responsible for restricting viral entry. The only site with significantly different editing between the two ZIKV strains occurred in the NCK2 gene. NCK2 is an adapter for receptor tyrosine kinases believed to be involved in cytoskeletal reorganization.

### Editing at miRNA binding sites

While a number of protein-recoding editing changes were observed, the majority of changing editing sites occurred in 3′ UTR regions. This is significant because of the 3′ UTR’s role in regulating gene translation and expression, including through the binding of miRNAs [70]. Given previous reports that ADAR editing can affect miRNA binding [35, 48, 71], we evaluated whether editing sites disrupted by congenital infection may have effects on gene expression by altering miRNA binding. After identifying differentially genes known to be targeted by miRNAs using TarBase [72], SubmiRine [73] was used to evaluate differences in miRNA binding between unedited and edited transcripts. While no editing was found to alter miRNA binding in the MCMV or hiNPC ZIKV datasets, editing sites with potential links to changes in miRNA targeting were detected in 2 genes for the mouse ZIKV PRJNA487357 dataset and 26 genes for the mouse ZIKV PRJNA358758 dataset. Importantly, a number of these genes had links to neurological disease, development, and function (Supplemental File 11).

## Discussion

Congenital infections commonly cause neurodevelopmental abnormalities through poorly understood mechanisms, often implicating the immune response. Here, we raised the question of whether the IFN-driven induction of ADAR RNA editing enzyme expression affects editing of host transcriptome, and whether such editing dysregulation may be related to the neurodevelopmental abnormalities caused by congenital infection [54]. Our RNA-seq data analyses provide evidence for changes in ADAR expression and editing caused by congenital infection by CMV and ZIKV in mice, and to a lesser degree, ZIKV infection of hiNPCs. In all cases, hyperediting of repetitive regions was increased in infected samples, and all samples showed a number of site-specific editing changes as well, with diverse functional implications. Crucially, our findings show changes in editing in genes relevant to neural development and function, providing a potential link to the neurodevelopmental abnormalities caused by congenital infection.

Interestingly, while our findings show increased hyperediting of repetitive regions across all datasets, site-specific editing changes tended to be much more heterogenous. This is consistent with previous studies in suggesting a role for a set of unknown highly sensitive factors in both host and virus that may regulate changes in transcriptome-wide ADAR editing landscapes [23, 74]. For example, the changes in ADAR2 and 3 expression observed specifically in the PRJNA358758 mouse ZIKV dataset could influence editing rates in unique ways compared to the other datasets with only ADAR1 overexpression.

Moreover, while it is difficult to compare between human and mouse results due to the lack of clearly orthologous sites, there was a high degree of overlap between differentially edited sites between mouse datasets. Nine of 12 editing sites dysregulated in the mouse ZIKV PRJNA487357 dataset and 13 of 21 dysregulated sites in the MCMV dataset were also dysregulated in the mouse ZIKV PRJNA358758 dataset, and 3 editing sites were dysregulated in all 3 datasets. All sites observed changed in the same direction for each dataset, indicating a set of consistently changing editing sites during infection. We were also interested in whether an expression of a specific gene may impact changes in RNA editing, for example, if overexpression of IFN-stimulated genes could impact their editing, leading to heterogeneity based on host and virus specific factors. However, we found no clear relationship between these variables (Supplementary Fig. 2).

One of the most significant editing sites that showed differential editing was the AMPA glutamate receptor subunit GRIA3 R/G recoding site, which was consistently underedited in ZIKV-infected samples for the PRJNA358758 dataset. Normally, editing of this site displays a gradual increase in editing through development [75]. The resulting R/G recoding causes faster recovery from desensitization, allowing quicker responses to impulses [76]. This finding reinforces previous evidence of altered GRIA3 editing caused by ZIKV infection [54]. Another significant neurological target that showed differential editing (increased) in the PRJNA358758 dataset was the GRIK5 K/R recoding site. GRIK5 is another excitatory glutamate receptor for which reduced expression has been associated with eye and vascular disease [77], and whose variants have been associated with neurological disorders [78]. While this gene has been shown to be consistently edited, the effect of K/R recoding on protein function and neurological phenotype has not been fully elucidated [79, 80].

Another one of the primary neurological genes found here was Calm1, edited in both PRJNA487357 and PRJNA358758 mouse ZIKV datasets. Calm1 regulates significant neurological functions, such as long-term potentiation (LTP), a mechanism for synaptic plasticity relevant to learning and memory [81] and smooth muscle contraction [82]. Other genes in the calmodulin pathway were also found to be differentially edited, including calmodulin-dependent kinase IV (Camk4) and Map6 (STOP), which saw altered editing in the PRJNA358758 mouse ZIKV dataset. Camk4 regulates synaptic excitation [83], memory formation [84], protects neurons from apoptosis [85], and has been implicated in neurodevelopmental disease [86]. Additionally, Map6 stabilizes microtubules and is targeted to the axons of polarizing neurons, playing an important role in axon maturation and the establishment of polarity [87], and has been linked to defects in neurotransmission and synapse formation leading to cognitive and behavioral impairments [88–94]. Interestingly, several 3′ UTR sites in Lamp2 saw dysregulated editing in MCMV-infected microglia. Previous studies have linked dysregulation of APOBEC C-to-T RNA editing of the Lamp2 3′ UTR in mouse microglia to lysosomal dysfunction, and subsequent neurological dysregulation and neurodegeneration [95]. In light of our finding of dysregulation of ADAR editing in the APOBEC1 gene, this result shows a potential connection between dysregulated editing in our dataset and neurological phenotypes in mice.

Notably, differential editing occurred in several genes responsible for regulation of mRNA splicing. The most significant of these was the Nova1 S/G recoding site (overedited in PRJNA358758), but also included 3′ UTR editing sites in genes such as spliceosome subcomponent Sf3b2 (overedited in all mouse datasets) and RNA binding protein Celf1 (overedited in PRJNA358758). Dynamic regulation of alternative splicing plays an important role in brain development, and dysregulation of this process has implications for neurodevelopmental disorders [96–98]. Specifically, Nova1 is a neurologically expressed RNA binding protein responsible for regulating alternative splicing relevant to synaptic function [97, 98]. Nova1-knockout mice experience neuronal apoptosis in the brain stem and spinal cord, followed by motor dysfunction and postnatal death [97, 98]. ADAR editing and S/G recoding of Nova1 is highly evolutionarily conserved, dynamically regulated with increasing levels through brain development, and has been shown to increase Nova1 protein stability by protecting against proteasomal degradation [99]. In doing so, dysregulation of this editing site has been implicated in neurological disease in both humans and mice [100]. Indeed, in PRJNA358758 where Nova1 S/G editing was found to be dysregulated, alternative splicing of a Nova targets was detected, including in 13 disease-associated genes.

Another area of interest is the potential disruption of epigenetic genome regulation. Phc2 (also known as Mhp2), a component of the class II Polycomb gene (PcG) complex as well as the Polycomb repressive complex 1 (PRC1) [101] has differential editing in the 3′ UTR region, which may alter miRNA targeting, in the PRJNA358758 and PRJNA487357 mouse ZIKV datasets. Disruption of class II PcG genes alters the expression of Hox cluster genes in the paraxial mesoderm and neural tube and causes axial skeleton malformations [102]. Phc2 is expressed in NPCs [103] and represses the expression of neurogenic genes during later stages [104]. This illustrates an interesting possibility by which editing dysregulation could disrupt neurodevelopment.

The analysis of hiNPC data revealed a different set of differentially edited sites. Two differentially edited genes are associated with neurodevelopmental disorders. An intronic site in the NCK2 was overedited in cells infected with ZIKV PE243V compared to the FSS1302 strain, while another intronic site in the B9D1 gene was unedited in the ZIKV FSS1302 strain, but highly edited in controls. NCK2 is a tyrosine kinase adaptor responsible for regulating cytoskeleton organization and may thusly contribute to the formation of proper neuronal connections [105–107]. B9D1 is important for ciliogenesis and has been implicated in ciliopathies including Meckel syndrome [68, 69], characterized by renal cystic dysplasia and CNS defects, and Joubert syndrome [108–110], characterized by cerebellar and brainstem malformation.

However, a number of potentially significant editing changes were also observed in immune genes for the hiNPC dataset. First, an exonic V/A recoding site in the IFITM2 gene was overedited in cells infected with both strains of ZIKV compared to controls. IFITM2 is an interferon-stimulated transmembrane protein which restricts viral membrane fusion and entry of many viruses including ZIKV [111–113]. While the impact of this recoding site on IFITM2 protein function is currently unknown, this might mean that changes in ADAR editing could influence the efficiency of ZIKV entry. Additionally, three sites in the 3′ UTR of DDX58 (RIG1) were overedited in the ZIKV FSS1302 strain compared to controls. RIG1 is an RNA helicase and dsRNA sensor critical for inducing the antiviral type 1 IFN response. Taken together, these could indicate that ADAR editing could modulate other aspects of the interferon response.

Aside from these, many other sites were impacted by differential ADAR editing, with some notable highlights described in Table 2. Future studies could use sites listed here as candidates to validate the neurodevelopmental effects of virus-induced editing dysregulation. Many neurological genes with dysregulated editing could contribute to death of neuronal cells, disrupt the formation of neuronal connections or lead to altered synaptic transmission. Disruption of other genes such as those regulating splicing programs or epigenetic gene silencing could alter regulatory programs in NPCs at a crucial point in their development. In addition, the immunological targets with differential editing could lead to a dysregulated immune response, which could have adverse effects through increased susceptibility to viral infection or immune-mediated damage or alterations to neurons or NPCs.

**Table 2:** List of genes with significant editing dysregulation, namely, observed differential editing in exonic regions with effects on protein-coding regions, in neurologically or immune relevant pathways, or in multiple samples. Parenthetical numbers next to a dataset for a given gene specify that > 1 site was differentially edited in that gene/dataset

Gene	Dataset(s)	Genic location of edited site/Effect	Function/significance of edited gene
**Neurological:**			
Gria3	PRJNA358758	Exon (nonsyn)	AMPA glutamate receptor subunit, editing allows faster recovery from desensitization [76]
Grik5	PRJNA358758	Exon (nonsyn)	Kainate glutamate receptor subunit associated with psychiatric, eye, and vascular diseases [77, 78]
Calm1	PRJNA358758, PRJNA487357	3′ UTR	Ca2 + −binding messenger impacting numerous neurological functions [81, 114, 115]
Camk4	PRJNA358758	3′ UTR	Signal transducer downstream of Calmodulin with important and diverse neurological and immune functions [116]
Map6	PRJNA358758	3′ UTR	Calmodulin binding protein regulating microtubule stability, important for proper axon development, disruption leads to issues with neurotransmission and synapse formation, as well as cognitive/behavioral deficits [87–94]
Selenot	PRJNA358758 (2), PRJNA487357	3′ UTR	Thioredoxin-like oxidoreductase, neuroprotective, highly expressed during brain development, KO affects brain structure through neuron loss and causes behavioral changes, important role in brain development [117]
Sgpl1	PRJNA358758, PRJEB38849	3′ UTR	Sphingosine phosphate lyase, regulates neuronal autophagy [118], microglial autophagy and inflammation [119], mutations linked with neurological pathologies [120–122]
Arhgdia	PRJNA358758, PRJEB38849 (2)	3′ UTR	Regulator of Rho GTPase signaling, regulates cell proliferation and migration, underexpression promotes glioma progression [123, 124]; Rho GTPase signaling plays an important role in neurodevelopment and dysregulation may lead to neurological disorders [125]
NCK2	PRJNA551246	Intron	Tyrosine kinase adaptor protein regulating cytoskeleton organization and formation of neuronal connections [105–107]
**Immune:**			
Lgals3	PRJNA358758	Exon (nonsyn)	Galectin with affinity for beta-galactosides, can regulate adhesion/inflammation of immune cells, can affect cell growth/differentiation, including immune cell/neurite growth, and acts as a splicing factor along with other functions [126–128]
Tapbp	PRJNA358758 (3), PRJNA487357 (2), PRJEB38849 (2)	3′ UTR	Mediates interaction between MHC1 and TAP to allow loading of antigenic peptides [129]
Xbp1	PRJNA358758, PRJEB38849	3′ UTR	Transcription factor regulating immune and UPR functions, also with links to neurodegenerative disease [130]
Ube2d3	PRJNA358758	3′ UTR	E2 ubiquitin ligase, involved in RIG-1 activation [131]
RIG1	PRJNA551246 (3)	3′ UTR	dsRNA sensor responsible for activating innate antiviral type 1 interferon response [132]
IFITM2	PRJNA551246	Exon (nonsyn)	IFN-stimulated antiviral restriction factor [111–113]
**Other:**			
Ucp2	PRJNA358758	Exon (nonsyn)	Mitochondrial uncoupling protein (proton leak), attenuates mitochondrial ROS production. Reduced expression is linked to altered differentiation of NPCs and has a significant role in brain development [133, 134]
Ogdh	PRJNA358758	Exon (stoploss)	2-oxoglutarate dehydrogenase complex subunit, some evidence of links to neurological disease [135, 136]
Azin1	PRJNA358758	Exon (nonsyn)	Antizyme inhibitor regulating intracellular polyamine levels, ADAR editing of this gene is linked to development of a number of cancers, including colorectal, non-small-cell lung, gastric, and hepatocellular cancers [137–142], as well as to hematopoietic stem cell differentiation [143]
Nova1	PRJNA358758	Exon (nonsyn)	RBP regulating splicing and degradation of a number of genes with important neurological functions through brain development: editing is dynamically regulated during development and increases protein stability [97–99]
Celf1	PRJNA358758	3′ UTR	RBP regulating splicing during brain development [144]
Sf3b2	PRJNA358758, PRJNA487357, PRJEB38849	3′ UTR	Splicing factor, U2 snRNP component, variants associated with craniofacial microsomia [145]
Sept2	PRJNA358758, PRJNA487357, PRJEB38849	3′ UTR	Cytoskeletal GTP-binding filament-forming protein Sept2 [146] is expressed in the brain, and neurological functions include regulation of astrocyte glutamate uptake [147]
Lamp2	PRJNA358758 (3), PRJEB38849 (3)	3′ UTR	Lysosome membrane glycoprotein, dysregulation of RNA editing by APOBEC1 in this gene in mouse microglia causes neurological dysfunction and neurodegeneration [95, 148]
Phc2	PRJNA358758, PRJNA487357	3′ UTR	Component of class II PcG complex and PRC1, contributes to epigenetic regulation of gene expression, including neurogenic genes during brain development [101–104]
H19	PRJNA358758, PRJNA487357	lncRNA	lncRNA which functions as a tumor suppressor and regulates growth during embryonic development [149, 150]
Gpx3	PRJNA358758, PRJEB38849	3′ UTR	Glutathione peroxidase, reduces hydrogen peroxide to prevent oxidative damage [151]
Fam49b (a.k.a. Cyrib)	PRJNA358758, PRJEB38849	Exon (syn)	Interacts with Rac GTPase, functions include mitochondrial ROS suppression, modulating cytoskeleton organization, and inhibition of T cell activation [152, 153]
Tmem50b	PRJNA358758, PRJEB38849	3′ UTR	Transmembrane protein, ER localization, may contribute to proper brain development, with dysregulation leading to Down syndrome-related phenotypes [154]
Cap1	PRJNA358758	3′ UTR	Regulates cytoskeleton organization, adhesion, cAMP signaling [155]. Also plays a role in regulating neuron differentiation [156, 157]

These editing changes were observed in a diverse range of biological contexts, including mouse microglia in the MCMV dataset, whole mouse brains for the ZIKV datasets, and human cell culture with hiNPCs. However, each dataset comes with their limitations, including the lack of data on other cell types for MCMV, and the limitations inherent to cell culture models for the ZIKV hiNPC dataset. In addition, the bulk brain tissue data for mouse ZIKV infections obscures insights into the potential brain region and cell type-specific regulation of editing [158, 159]. The potential pitfalls of inferring editing changes from whole brain sequencing are shown by [160], that found presumed changes in editing following spinal cord injury inferred by others, such as [161], were simply due to decreased neuron density, as neurons have different editing rates than other CNS cell populations. In addition, while the HCMV microarray data contains no information about editing, it shows that viral-induced consistently increased ADAR expression patterns are recapitulated in large, human, clinical datasets of congenital infections, including in asymptomatic cases. This demonstrates the potential clinical significance of these phenomena and the importance of further investigation of editing in this context. When comparing across diverse datasets, it is possible that differences in experimental procedures used in these studies may be responsible for observed differences between datasets. For example, differences in viral delivery method and viral load (as summarized in Table 1) could lead to differences in immune responses or patterns of infection that, in turn, influence ADAR editing. In addition, differences in the developmental stages at which infection and sequencing occurred could further contribute to differences between samples. Given the dynamic regulation of ADAR editing through development [162], as well as general changes in transcriptome composition over time, interventions at different stages may have different effects on editing. The length of time between infection and sample collection could also impact editing changes observed, especially in light of the work of [56] demonstrating the transient nature of MIA-induced editing changes. Another critical factor that may result in editing variations is biological sex, which can result in significant differences in editing patterns and was not examined here.

There are a number of other technical limitations to our findings from the data analyzed here. First, it is worth noting that the number of editing sites found here was relatively small for some datasets, potentially owing to a lack of depth in sequencing. In particular, sequencing depth ranged from 16to 25 million reads in PRJEB38849 MCMV samples and 10 to 16 million reads for PRJNA487357 ZIKV samples, whereas for paired end Illumina sequencing, 80–100 million reads is generally desirable for RNA editing detection [163]. Future studies using high-depth sequencing would be helpful to fully illuminate the range of editing dysregulation caused by congenital infection. The benefits of this approach are illustrated by [56] analysis of MIA-induced RNA editing changes, where experiment repetition with 220 million read coverage revealed more robust changes in RNA editing and new dysregulated editing sites. Additionally, all RNA-seq datasets used here had three samples per condition, which limits the extent to which these findings can be extrapolated to the broader population.

Another serious limitation of our study is the inability to examine potential editing-independent effects of changes in ADAR expression. ADAR can have many cellular effects through direct interactions with other proteins and competitive binding to RNA. First, in the immune response, ADAR1 can bind to and inhibit the activity of PKR, an IFN-induced gene which plays a role blocking translation as part of the antiviral immune response [53, 164]. In addition, ADAR affects a number of RNA processing pathways independent of editing. For example, ADAR1 can form a complex with miRNA processing protein Dicer to promote the rate of pre-miRNA cleavage and formation/loading of the RISC complex [26, 45–47]. ADAR2 can also regulate splicing independent of editing by competing with U2AF65 for 3′ splice site binding. Other editing-independent effects of ADAR include regulation of gene expression through interactions with HuR [71] and NF90 [165]. Given that ADAR expression was drastically altered in samples with viral infections, many of these processes may be altered in ways that cannot be linked to editing. Future studies should explore this possibility.

The evidence presented here is consistent with the hypothesis that congenital CMV and ZIKV infection induces changes in ADAR editing, which in turn disrupts brain development. However, a causal link between virus-induced RNA editing dysregulation of specific transcripts and neurodevelopmental symptoms remains to be established. A useful first step here would be to test the effects of congenital infection with knock-downs or inhibition of ADAR and/or IFNs at different times during fetal development. This could help determine what role, if any, ADAR has in the development of specific neurodevelopmental symptoms. It may also be useful to test the effects of congenital infection in mice/cells with editing-inactive ADAR enzymes. This could help delineate what effects of ADAR are due to editing, and which may be due to ADAR interactions with other proteins. Following this, the specific editing sites/interactions that result in neurodevelopmental abnormalities could be probed. This may involve testing the effects of specific editing sites by investigating brain development in the presence of different RNA variants or different combinations of RNA variants, or investigating the activity of ADAR binding partners. These factors should be tested in different cell types and brain regions, and at different developmental stages. This would give us a much more granular, mechanistic understanding of the relevance of ADAR editing during congenital infection in the brain.

## Conclusions

Overall, our results support the hypothesis that congenital viral infections by ZIKV and CMV induce expression of ADAR1 and disrupt normal host transcriptome regulation during brain development. This has significant implications for understanding the mechanisms behind the pathogenesis of neurodevelopmental sequelae of congenital viral infections. We also lay out further experimentation that is necessary to confirm this hypothesis and to give a full mechanistic understanding of the effects of ADAR during congenital infection. We also suggest that future research should elucidate editing patterns caused by congenital infection of other viruses, given the highly virus-dependent nature of editing changes. This would be useful to inform potential novel treatment pathways, and to better understand the risks viral infection can pose to the developing brain.

## Methods

### BioProject datasets

Data from BioProjects PRJNA422858, PRJEB38849, PRJNA487357, PRJNA358758, and PRJNA551246 were used. BioProject PRJNA422858 contains microarray expression data from blood samples of infants with asymptomatic and symptomatic human CMV (HCMV) infections, and healthy controls. BioProject PRJEB38849 contains RNA-seq data from newborn mouse microglia, 3 with mouse CMV (MCMV) infection, and 3 controls. BioProject PRJNA487357 and PRJNA358758 contain RNA-seq data from fetal mouse brain tissues, 3 infected with ZIKV, and 3 controls. And PRJNA551246 contains human induced pluripotent neuroprogenitor stem cells (hiNPCs), 3 infected with the Cambodian strain of ZIKV (FSS13025), and 3 infected with the Brazilian strain of ZIKV (PE243V), and 3 controls.

### Microarray data analysis

For BioProject dataset PRJNA422858/GEO dataset GSE108211 (HCMV), GEO2R (https://www.ncbi.nlm.nih.gov/geo/geo2r/) [58] was used to evaluate differential gene expression, corrected for multiple hypothesis testing (FDR, false discovery rate) with the Benjamini–Hochberg procedure [166], quantile normalization and log2 transformation as implemented in GEO2R, with a specific focus on ADAR expression. Reactome [59] was then used to identify overrepresentation of differentially expressed immune pathways that may be related to the changes in ADAR expression. However, because the dataset only contains microarray gene expression data, the extent of editing could not be evaluated for these samples.

### RNA-seq data analysis: variant calling and identification of editing sites

Next, the RNA-seq datasets were analyzed for ADAR expression and editing. For these samples, variant frequency counts produced by the Automated Isoform Diversity Detector (AIDD) pipeline [167] were used to evaluate the extent of RNA editing. Briefly, AIDD used HISAT2 [168] for genome alignment with the GRCm38/mm10 reference genome for mouse samples and the hg37 reference genome for human samples, followed by StringTie [169] for genome assembly and transcript counting as transcripts per million (TPM). GATK HaplotypeCaller [170] was then used for variant calling to identify ADAR edited sites. Bam-readcount [171] was used to determine the number of individual bases observed at each potentially edited site. To ensure that we are not including false positives, we further defined editing sites as those variants that occur in the REDIportal database V2.0 [172] with a reference of A or T (to identify A-to-G sites, or T-to-C as the nucleotide change would be interpreted on the opposite strand), with greater than 3 total reads, and with an editing rate (defined as the percent of G reads for an A reference sites or C reads for a T reference site) greater than 0.01, less than 0.99, and not between 0.49 and 0.51 (to remove potential noise, homozygous genomic variants, and heterozygous genomic variants, respectively).

Presence of viral reads in the infected samples was verified by mapping reads remaining unmapped to the respective viral genomes of MCMV and ZIKV using the following reference genomes: NC_075725 (MCMV), KX520666 (ZIKV1), KU866423 (ZIKV2), and KX197192 and MH158236 for human ZIKV dataset, respectively. As expected, viral reads represented only small portions of unmapped reads in the infected samples, with uninfected samples harboring essentially zero viral reads. This allowed us to confirm the infection status of respective samples. On average, there were ~ 0.76% (out of total unmapped reads) viral reads detected in MCMV samples, ~ 2.1% for mouse ZIKV1, and ~ 23.2% for mouse ZIKV2 and human ZIKV samples, respectively (Supplementary File 12).

### Statistical analysis of RNA-seq editing and expression data

DESeq2 [61] was used to conduct differential expression analysis to examine changes in expression for ADAR enzymes. Additionally, T-tests (pairwise in the case of hiNPC data) were performed on editing rates and corrected for multiple hypothesis testing with the Benjamini–Hochberg procedure. Significance was determined with a threshold of 0.05. To determine changes in global levels of RNA editing, we used the *Alu* editing indexing (AEI) method [62], which takes the ratio of A-to-G mismatches to the total coverage of As in hyperedited repetitive elements: B1/B2 elements in mice, and *Alu* elements in humans (analogous to the percentage of A-to-G editing in these regions). Finally, Reactome pathway overrepresentation analysis was performed for editing sites with significant differences in editing.

### Analysis of the effects of editing on miRNA binding

Sequences of 3′ UTRs with differentially edited sites were obtained from Ensembl Biomart [173], and editing coordinates were converted to their GRCm39 equivalents using CrossMap [174]. This step was used to generate edited versus wild type sequences for each edited gene/site with available sequences for all transcript isoforms containing the given coordinate. TarBase [72] was used to identify genes known to be targeted by miRNAs, and miRNA sequences were obtained from miRBase [175]. Finally, these sequences were used to find differences in miRNA targeting between edited and unedited transcripts using SubmiRine [73].

### Analysis of differential splicing

MAJIQ and VOILA software packages (Vaquero-Garcia et al., 2023) were used to assess changes in alternative splicing between infected and control samples in PRJNA358758 (ZIKV). MAJIQ builder constructed splice graphs, and MAJIQ quantifier was used to quantify percent spliced in (PSI) and delta PSI (dPSI) of local splicing variations (LSVs). VOILA was used to output LSVs with P(|dPSI| > 0.2) > 0.95.

### Supplementary Information

**Additional file 1: Supplementary Figure 1.** Violin and box plots showing the distribution of RNA editing rates of sites detected in each sample. Panels A-C show editing rate distributions in viral infection (blue) and control (red samples) for MCMV, ZIKV PRJNA487357, and ZIKV PRJNA358758 samples, respectively, Panel D shows editing rate distributions in ZIKV FSS13205 (green), ZIKV PE243 (blue), and control (red) hiNPC samples.

**Additional file 2: Supplementary Figure 2.** Scatterplots of correlations between changes in expression (TPM) and changes in RNA editing rates. (A) Plot of change in TPM and editing rate for each editing sate between MCMV and control samples (R^2 = 0.01932). (B) Plot of change in TPM and editing rate for each editing sate between ZIKV and control samples for PRJNA487357 (R^2 = 0.03809). (C) Plot of change in TPM and editing rate for each editing sate between ZIKV and control samples for PRJNA 358758 (R^2 = -0.001244). (D) Plot of change in TPM and editing rate for each editing sate between ZIKV PE243 and control hiNPC samples (R^2 = 0.07111). (E) Plot of change in TPM and editing rate for each editing sate between ZIKV FSS13205 and control hiNPC samples (R^2 = 0.05415). (F) Plot of change in TPM and editing rate for each editing sate between ZIKV FSS13205 and PE243 samples (R^2 = -0.0054).

**Additional file 3: Supplementary File 1.** Lists of differentially expressed genes (DEGs) from comparisons of symptomatic and asymptomatic HCMV infections to control samples, from GEO2R analysis of BioProject dataset PRJNA422858 (https://www.ncbi.nlm.nih.gov/geo/geo2r/?acc=GSE108211). Sheets A, B and C show GEO2R lists of DEGs from symptomatic vs control, asymptomatic vs control, and symptomatic vs asymptomatic HCMV samples comparisons. Sheets D and E show results of Reactome pathways overrepresentation analyses for significant (FDR <= 0.05) DEGs from symptomatic vs control and asymptomatic vs control HCMV samples. Only pathways with entities FDR < 0.05 are shown.

**Additional file 4: Supplementary File 2.** List of DESeq2 (Love et al., 2014) differential expression analysis results for gene counts from ballgown, including log2 fold change values and p values for expression changes in each gene for (A) MCMV vs control, (B) ZIKV vs control (PRJNA487357), (C) ZIKV vs control (PRJNA358758), (D) ZIKV FSS13205 vs control hiNPC, (E) ZIKV PE243 vs control hiNPC, and (F) ZIKV PE243 vs FSS13205 samples.

**Additional file 5: Supplementary File 3.** List of DESeq2 (Love et al., 2014) differential expression analysis results for transcript counts from ballgown, including log2 fold change values and p values for expression changes in each transcript for (A) MCMV vs control, (B) ZIKV vs control (PRJNA487357), (C) ZIKV vs control (PRJNA358758), (D) ZIKV FSS13205 vs control hiNPC, (E) ZIKV PE243 vs control hiNPC, and (F) ZIKV PE243 vs FSS13205 samples.

**Additional file 6: Supplementary File 4.** Alu editing index (AEI) values from the RNA Editing Indexer method (Roth et al., 2019) for (A) the PRJEB38849 MCMV dataset, (B) the ZIKV PRJNA487357 dataset, (C) the ZIKV PRJNA358758 dataset, and (D) the hiNPC ZIKV PRJNA551246 dataset. This includes quantifications of the ratio of A-to-G mismatches to total A reads (effectively the percent of As edited to Gs) in *Alu* repeat elements in humans or SINE B1/B2 repeat elements in mice, as well as the same metric for other variant types. A-to-G editing index serves as a general metric of transcriptome-wide levels of hyperediting, which primarily occur in repeat regions. Quantification of other variants serve as a measure of background noise, with the next most common modification type being C-to-T editing.

**Additional file 7: Supplementary File 5.** (A) Characteristics of 149 editing sites from MCMV and control samples. List of ADAR edited sites (identified via chromosome (CHR) and position (POS)) and individual nucleotide counts from MCMV infections and control samples (PRJEB38849). (B) Characteristics of 21 significantly different editing sites between MCMV and control samples. List of ADAR edited sites (identified via chromosome (CHR) and position (POS)) and average editing rates from MCMV.

**Additional file 8: Supplementary File 6.** Reactome pathway analysis of edited genes from infected and control samples. In bold are pathways overrepresented among editing targets with FDR < 0.1. (A) Sheet 6A shows overrepresented pathways among edited targets from MCMV and control samples (PRJEB38849). (B) Sheet 6B shows overrepresented pathways among edited targets from ZIKV and control samples (PRJNA487357). (C) Sheet 6C shows pathways among edited targets from ZIKV and control samples (PRJNA358758); there were no pathways overrepresented among editing targets with FDR < 0.1.

**Additional file 9: Supplementary File 7.** (A) Characteristics of 78 editing sites from ZIKV and control samples (PRJNA487357). List of ADAR edited sites (identified via chromosome (CHR) and position (POS)) and individual nucleotide counts from ZIKV infections and control samples. (B) Characteristics of 12 significantly different editing sites between ZIKV and control samples. List of ADAR edited sites (identified via chromosome (CHR) and position (POS)), and average editing rates from ZIKV infections and control samples (PRJNA487357).

**Additional file 10: Supplementary File 8.** (A) Characteristics of 1276 editing sites from ZIKV and control samples. List of ADAR edited sites (identified via chromosome (CHR) and position (POS)) and individual nucleotide counts from ZIKV infections and control samples (PRJNA358758). (B) Characteristics of 148 significantly different editing sites between ZIKV and control samples. List of ADAR edited sites (identified via chromosome (CHR) and position (POS)) and average editing rates from ZIKV infections and control samples (PRJNA358758).

**Additional file 11: Supplementary File 9.** Results of MAJIQ and VOILA analysis of editing sites from ZIKV and control samples (PRJNA358758). (A) Results of MAJIQ analysis of editing sites from ZIKV and control samples (PRJNA358758), with PSI/dPSI information for all LSVs identified. (B) Results of VOILA analysis of editing sites from ZIKV and control samples (PRJNA358758), with significant LSVs (|dPSI| > 0.2 and p < 0.05). (C) Information for LSVs in genes identified as Nova1 targets (Zhang et al., 2010).

**Additional file 12: Supplementary File 10.** (A) Characteristics of 1355 editing sites from ZIKV and control samples (PRJNA551246). List of ADAR edited sites (identified via chromosome (CHR) and position (POS)) and individual nucleotide counts from ZIKV infections with Cambodian (FSS13025) and Brazilian ZIKV (PE243) strains and control samples. (B) Characteristics of 9 significantly different editing sites between ZIKV and control samples (PRJNA551246). List of ADAR edited sites (identified via chromosome (CHR) and position (POS)), and average editing rates from ZIKV infections with Cambodian (FSS13025) and Brazilian ZIKV (PE243) strains and control samples.

**Additional file 13: Supplementary File 11.** SubmiRine (Maxwell et al., 2015) results predicting differences in miRNA binding between unedited and edited transcripts. While no editing was found to alter miRNA binding in the MCMV or hiNPC ZIKV datasets, editing sites with potential links to changes in miRNA targeting were detected in 2 genes for the mouse ZIKV PRJNA487357 dataset and 26 genes for the mouse ZIKV PRJNA358758 dataset.

**Additional file 14: Supplementary File 12.** Number of (and percent of unmapped) reads that were mapped to the respective viral genomes. Briefly, unmapped reads were collected from the BAM files and mapped to the viral genomes of MCMV and ZIKV using STAR. The following reference genomes were used: NC_075725 (MCMV), KX520666 (ZIKV1), KU866423 (ZIKV2), and KX197192 and MH158236* for human ZIKV dataset, respectively. As expected, the viral reads were found primarily in the infected samples, although their numbers represented only relatively small portions of all sequenced reads. A handful of viral reads were also detected in some uninfected samples, consistent with previously reported results (Lima et al. 2019, Fig. 4C) and the possibility of artifacts of mapping and/or negligible contamination. * MH158236 is a complete genome of JN860885 (FSS13025) ZIKV isolate.

## Data Availability

Supplementary Figures and Tables, with relevant input data files and R code, are available at GitHub repository at https://github.com/RNAdetective/Congenital_CMV-ZIKV_infections. The datasets used in this current study are publicly available in the NCBI SRA/BioProject repository, as BioProjects PRJNA422858, PRJEB38849, PRJNA487357, PRJNA358758 and PRJNA551246.

## Abbreviations

ADAR: Adenosine Deaminases Acting on RNA
CMV: cytomegalovirus
CNS: central nervous system
CZS: congenital Zika syndrome
FDR: false discovery rate
HCVM: human cytomegalovirus
hiNPCs: human induced pluripotent neuroprogenitor stem cells
IFN: interferon
ISRE: interferon-sensitive response element
LSVs: local splicing variations
LTP: long-term potentiation
MCMV: mouse cytomegalovirus
MIA: maternal immune activation
NPCs: neural progenitor cells
NSCs: neural stem cells
ReoV: reovirus
SNHL: sensorineural hearing loss
SREs: splicing regulatory elements
UTR: untranslated region
ZIKV: Zika virus
