# Supplementary figures and images for: Changes in ADAR RNA editing patterns in CMV and ZIKV congenital infections
Source: BMC Genomics. 2023 Nov 15;24:685. doi: 10.1186/s12864-023-09778-4 (PMC10652522; doi:10.1186/s12864-023-09778-4)

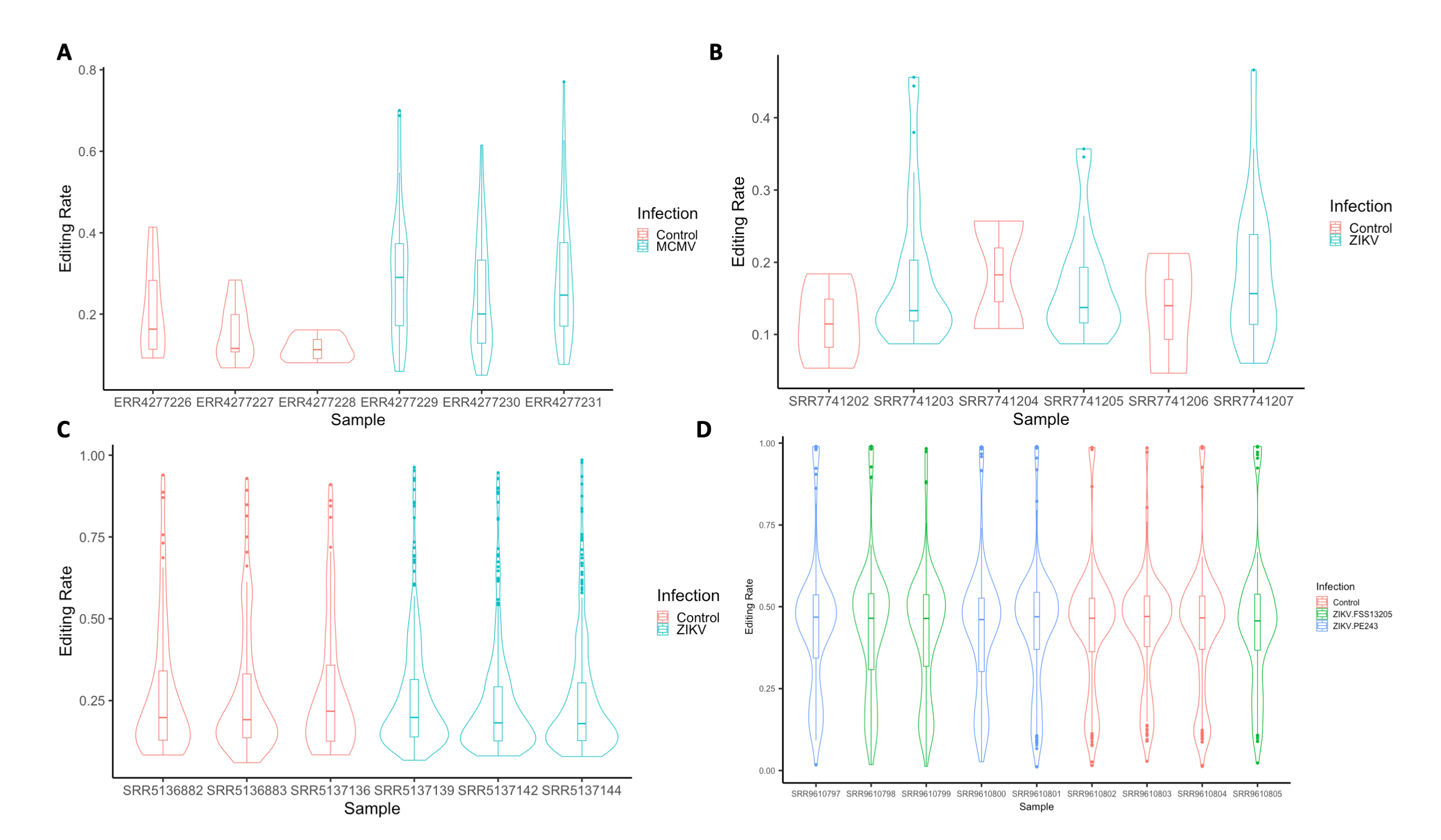

Supplement: Supplementary file 1 — Additional file 1: Supplementary Figure 1. Violin and box plots showing the distribution of RNA editing rates of sites detected in each sample. Panels A-C show editing rate distributions in viral infection (blue) and control (red samples) for MCMV, ZIKV PRJNA487357, and ZIKV PRJNA358758 samples, respectively, Panel D shows editing rate distributions in ZIKV FSS13205 (green), ZIKV PE243 (blue), and control (red) hiNPC samples. [file 12864_2023_9778_MOESM1_ESM.png]

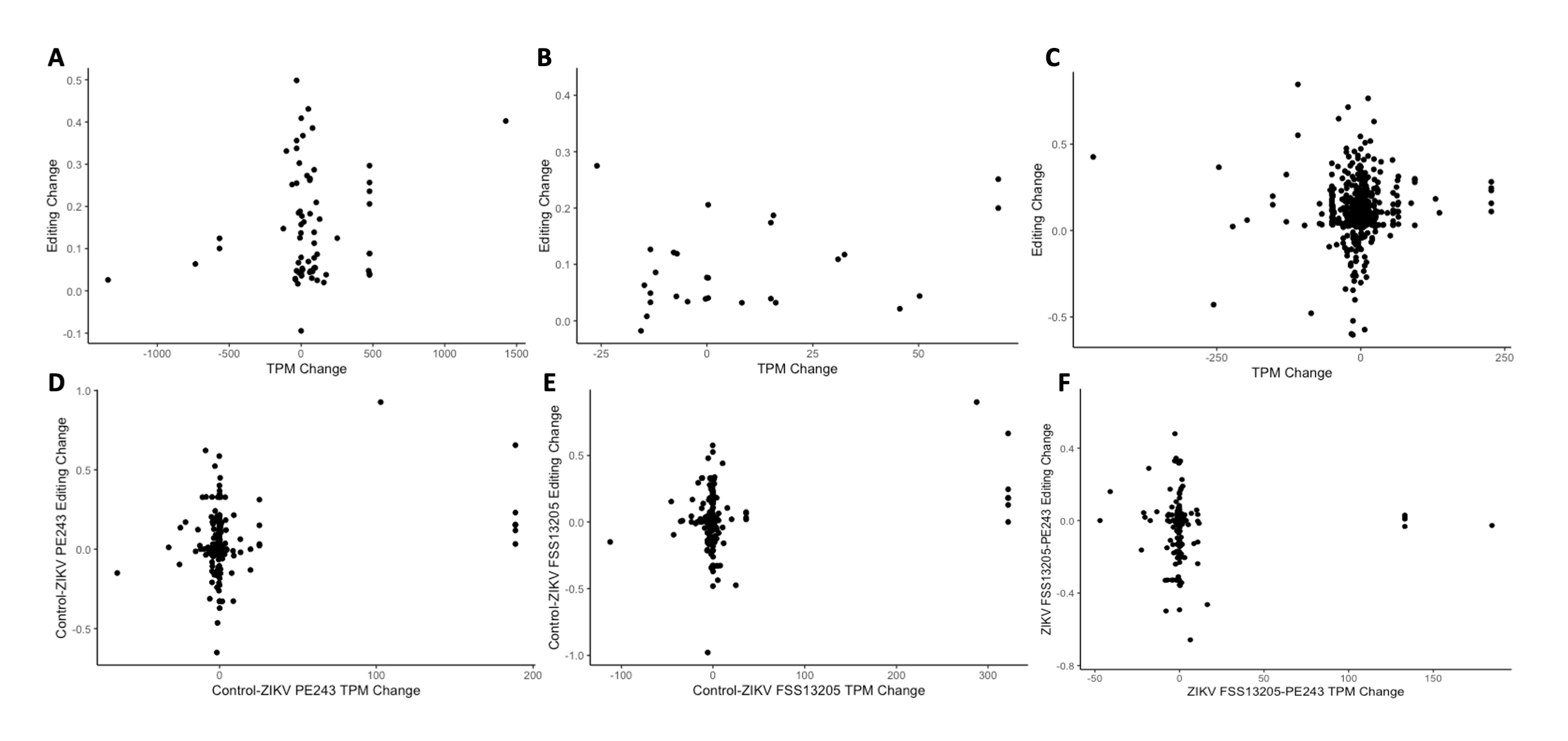

Supplement: Supplementary file 2 — Additional file 2: Supplementary Figure 2. Scatterplots of correlations between changes in expression (TPM) and changes in RNA editing rates. (A) Plot of change in TPM and editing rate for each editing sate between MCMV and control samples (R^2 = 0.01932). (B) Plot of change in TPM and editing rate for each editing sate between ZIKV and control samples for PRJNA487357 (R^2 = 0.03809). (C) Plot of change in TPM and editing rate for each editing sate between ZIKV and control samples for PRJNA 358758 (R^2 = -0.001244). (D) Plot of change in TPM and editing rate for each editing sate between ZIKV PE243 and control hiNPC samples (R^2 = 0.07111). (E) Plot of change in TPM and editing rate for each editing sate between ZIKV FSS13205 and control hiNPC samples (R^2 = 0.05415). (F) Plot of change in TPM and editing rate for each editing sate between ZIKV FSS13205 and PE243 samples (R^2 = -0.0054). [file 12864_2023_9778_MOESM2_ESM.png]
